# *Myrtus* Polyphenols, from Antioxidants to Anti-Inflammatory Molecules: Exploring a Network Involving Cytochromes P450 and Vitamin D

**DOI:** 10.3390/molecules24081515

**Published:** 2019-04-17

**Authors:** Sara Cruciani, Sara Santaniello, Giuseppe Garroni, Angela Fadda, Francesca Balzano, Emanuela Bellu, Giorgia Sarais, Giacomo Fais, Maurizio Mulas, Margherita Maioli

**Affiliations:** 1 Department of Biomedical Sciences, University of Sassari, Viale San Pietro 43/B, 07100 Sassari, Italy; sara.cruciani@outlook.com (S.C.); sara.santaniello@gmail.com (S.S.); giugarroni21@gmail.com (G.G.); mariafrancesca22@virgilio.it (F.B.); ema.bellu@hotmail.it (E.B.); 2 Institute of Sciences of Food Production (ISPA), Consiglio Nazionale delle Ricerche (CNR), Traversa la Crucca, 3, 07100 Sassari; angela.fadda@cnr.it; 3 Department of Life and Environmental Sciences, University of Cagliari, Via Ospedale 72, 09124 Cagliari, Italy; gsarais@unica.it (G.S.); giacomo.fais@unica.it (G.F.); 4 Department of Agriculture, University of Sassari, Via De Nicola 9, I-07100 Sassari, Italy; mmulas@uniss.it; 5 Center for Developmental Biology and Reprogramming- CEDEBIOR, Department of Biomedical Sciences, University of Sassari, Viale San Pietro 43/B, 07100 Sassari, Italy; 6 Istituto di Ricerca Genetica e Biomedica, Consiglio Nazionale delle Ricerche (CNR), 09042 Monserrato, Cagliari, Italy

**Keywords:** nutraceuticals, oxidative stress, inflammation, antioxidants, interleukins, CYPs expression, vitamin D

## Abstract

Inflammatory response represents one of the main mechanisms of healing and tissue function restoration. On the other hand, chronic inflammation leads to excessive secretion of pro-inflammatory cytokines involved in the onset of several diseases. Oxidative stress condition may contribute in worsening inflammatory state fall, increasing reactive oxygen species (ROS) production and cytokines release. Polyphenols can counteract inflammation and oxidative stress, modulating the release of toxic molecules and interacting with physiological defenses, such as cytochromes p450 enzymes. In this paper, we aimed at evaluating the anti-inflammatory properties of different concentrations of *Myrtus communis* L. pulp and seeds extracts, derived from liquor industrial production, on human fibroblasts. We determined ROS production after oxidative stress induction by H_2_O_2_ treatment, and the gene expression of different proinflammatory cytokines. We also analyzed the expression of CYP3A4 and CYP27B1 genes, in order to evaluate the capability of *Myrtus* polyphenols to influence the metabolic regulation of other molecules, including drugs, ROS, and vitamin D. Our results showed that *Myrtus* extracts exert a synergic effect with vitamin D in reducing inflammation and ROS production, protecting cells from oxidative stress damages. Moreover, the extracts modulate CYPs expression, preventing chronic inflammation and suggesting their use in development of new therapeutic formulations.

## 1. Introduction

Inflammation is a natural self-protecting mechanism developed by the organisms to remove harmful stimuli, such as damaged cells or pathogens, also being the initial step of healing processes and the restoration of tissue function [1]. Some pathologic conditions are related to inflammasomes dysregulation, inhibition of autophagy, or perturbations in ubiquitination [2,3]. Moreover, a persistent low-grade inflammation is considered one of the main causes of autoimmune and noncommunicable diseases [4,5,6,7]. The inflammasome is a multiprotein complex able to secrete proinflammatory cytokines, as Interleukin 1 beta (IL-1β) and Interleukin 18 (IL-18), upon activation by a stimulus, including for example UVB irradiations [8]. Cytokines production plays a key role in modulating the inflammatory response, especially during the acute phase [9,10]. Nevertheless, during the different steps of the inflammatory response, also other kind of cytokines, as tumor necrosis factor alpha (TNF-α), interleukin 6 (IL-6), and 12 (IL-12) are produced and released within a specific timeline [11,12].

Biochemical reactions in aerobic organisms involve reduction processes with the unavoidable production of reactive oxygen species. In normal conditions, physiological antioxidant defenses are able to counteract the damaging effects of ROS [13,14]. When the natural oxidation/reduction balance is impaired, a condition of oxidative stress is generated with a consequent alteration in P450 expression [15], as described in different kind of tumors [16,17]. Cytochromes P450 (CYPs) define a family of enzymes, expressed in liver and other tissues, including skin, playing a crucial role in the metabolism of different endogenous and exogenous molecules, for example, CYP27B1 for vitamin D3 [18,19,20]. The CYP3A4 is the most abundant P450-enzyme in the tissues, being responsible for the catabolism of more than 60% of drugs [21,22]. Its activity and expression can be modulated by a direct interaction with different molecules, for example, polyphenols [23,24,25]. Dysregulation in ROS production is also implicated in vascular injuries and atherosclerosis, and therapeutic approaches are actually based on antioxidant oral administration [26]. In addition, the interplay between oxidative stress and mitochondrial activity dysfunction can also affect the development and progression of Alzheimer and age-related diseases, cell function and senescence [27,28]. Several plants are known for their antioxidants content, which protect them from sun radiations, and at the same time, exert beneficial effects on oxidative stress in neurodegenerative diseases [29]. Recently, anticancer properties of polyphenols were described in different tumor cell lines [30,31,32]. Extracts from bulbs, leaves and flowers of blueberries and lingonberries for example, are rich in polyphenols and exhibit a high radical scavenging ability, suggesting their possible nutraceutical use as food supplements [33,34,35,36]. *Myrtus communis* L. is largely known in Mediterranean areas for the large number of flavonoids and galloyl derivatives presents in its berries and leafs. Antioxidant and anti-inflammatory activities of this plant are deeply documented [37,38]. In particular, its berries are used in popular medicine and in food industry for the liquor production [39]. Aim of the present study was to explore the effects of polyphenols present in *Myrtus communis* L. extracts, residual of the industrial production, on the expression of genes related to inflammation, in human skin fibroblasts (HFF1) exposed to oxidative stress. Oxidative stress, in fact, is considered at the moment as one of the causes of chronic inflammation. Thus, identifying novel sources of molecules able to counteract these processes could help for the design of novel anti-inflammatory preparations [40,41].

## 2. Results

### 2.1. Quantitative Data Analysis of Main Phenolic Compounds

Quantitative data analysis of main phenolic compounds identified in myrtle byproducts are reported in Table 1. Hydrolysable tannins, representing more than 90% on seed samples, dominated the phenolic profiles of both pulp and seeds. Phenolic acids were detected in lower amounts, characterized only by the presence of gallic acid (52.21 mg/kg dry weight DW for pulp and 137.02 for seeds). Among flavanols detected in fresh myrtle berries, quercetin-3-O-galactoside and, to a lesser extent, quercetin-3-O-rhamnoside, were the only flavanols detected. Quercetin-3-O-galactoside content of pulp (191.04 mg/kgDW) was twice that of the seeds extract (104.90 mg/kgDW). On the contrary, quercetin-3-O-rhamnoside was equally distributed in pulp and seeds samples with a concentration of 66.58 and 61.99 mg/kgDW, respectively. *Myrtus* by-products extracts act as a good source of ellagic acids present in larger amount in the seeds (726.94 mg/kgDW) than in the pulp (350.55 mg/kgDW). As expected, anthocyanins were detected only in pulp extract. According to industrial process, anthocyanins represent the smallest fraction of the entire extract with a malvidin 3-O-glucoside the most abundant (41.98 kgDW).

### 2.2. Myrtus Reduces ROS Production by H_2_O_2_

**Figure 1:**
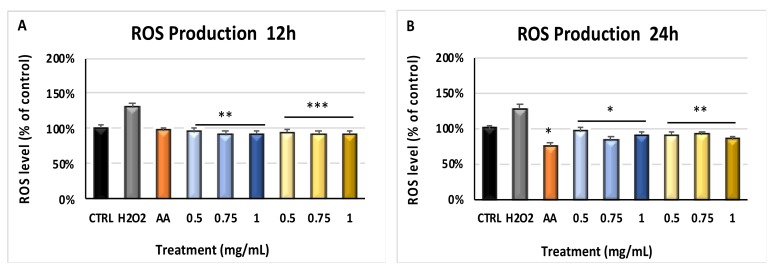
ROS levels after oxidative stress. The reactive oxygen species (ROS) production was evaluated in HFF1 exposed for 12 h (Panel (**A**)) or 24 h (Panel (**B**)) to ascorbic acid (AA, orange bar) or to 0.5, 0.75 and 1 mg/mL seeds (blue bars), or to 0.5, 0.75 and 1 mg/mL pulp (yellow bars) waste extracts, then induced to oxidative stress. “H_2_O_2_” (grey bar) represents HFF1 cells exposed to H_2_O_2_ alone, without a previous extracts-treatment. ROS levels of treated cells are expressed as a percentage of control untreated HFF1 (Ctrl, black bar), considered as 1. The concentrations were read as the absorbance at 529 nm emission wavelength for each sample and were expressed as mean ± SD referred to the control (* *p* ≤ 0.05, ** *p* ≤ 0.01 and *** *p* ≤ 0.001).

Figure 1 shows the amount of reactive oxygen species (ROS) in cells pretreated with pulp or seeds residuals, then exposed to oxidative stress. ROS production was significantly inhibited (*p* ≤ 0.01 and *p* ≤ 0.001) in cells pretreated with different concentrations of both pulp or seeds for 12 h (Panel A) and 24 h (*p* ≤ 0.5 and *p* ≤ 0.01) (Panel B), then exposed to H_2_O_2_, as compared to cells exposed to H_2_O_2_ without extracts pretreatment. The same figure shows that the antioxidant properties of residual extracts from industrial production is superimposable to the activities displayed by ascorbic acid, a compound well-known for its scavenging and anti-inflammatory activity [42].

### 2.3. Inflammatory-Related Gene Modulation by Myrtus Products

**Figure 2:**
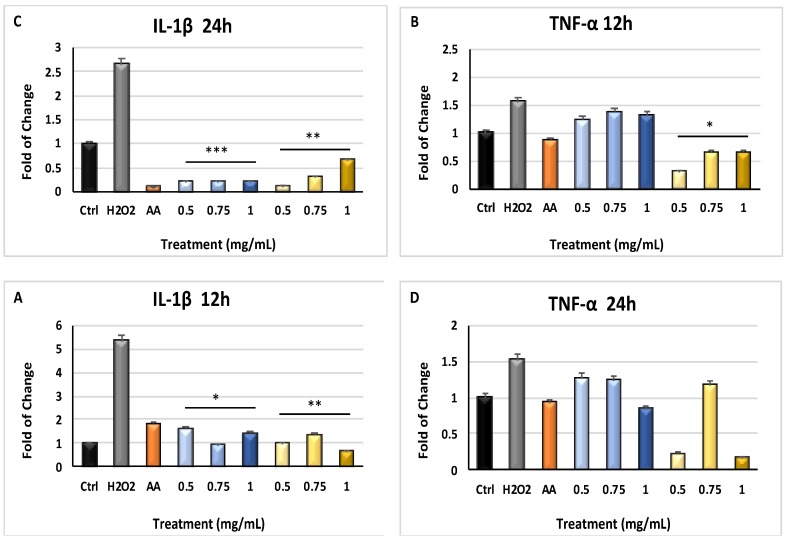
Gene expression of proinflammatory cytokines IL-1β and TNF-α. The expression of Interleukin 1 beta (IL-1β) and Tumor necrosis factor alpha (TNF-α) was evaluated in cells pre-treated with the extracts for 12 h and 24 h and then exposed to H_2_O_2_ (Panels (**A**) and (**B**), and panels (**C**) and (**D**), respectively). HFF1 were exposed to ascorbic acid (AA, orange bar), or to 0.5, 0.75 and 1 mg/mL seeds waste extracts (blue bars) or 0.5, 0.75 and 1 mg/ml pulp waste extracts (yellows bar). H_2_O_2_ (grey bar) represents HFF1 cells exposed to only H_2_O_2_, without previous extracts treatment. The mRNA levels for each gene was expressed as fold of change (2^−∆∆Ct^) of mRNA levels observed in untreated HFF1 (CTRL, black bar) defined as 1 (mean ±SD; n = 6) and normalized to Glyceraldehyde-3-Phosphate-Dehidrogenase (GAPDH). Data are represented as mean ± SD referred to the control (* *p* ≤ 0.05, ** *p* ≤ 0.01 and *** *p* ≤ 0.001).

Figure 2 shows that the mRNA levels of IL-1β (Panel A) and TNF-α (Panel B) were significantly downregulated (*p* ≤ 0.05 and *p* ≤ 0.01) in H_2_O_2_-treated cells which were previously exposed to both pulp or seeds extracts for 12 h, as compared to H_2_O_2_-treated cells not pre-exposed to the extracts. This effect was increased in cells cultured for 24 h in the presence of the extracts and then exposed to H_2_O_2_ (Panel C) (*p* ≤ 0.001).

### 2.4. Myrtus Extracts Act on Angiogenesis by Modulating Inflammatory Mediating Genes

**Figure 3:**
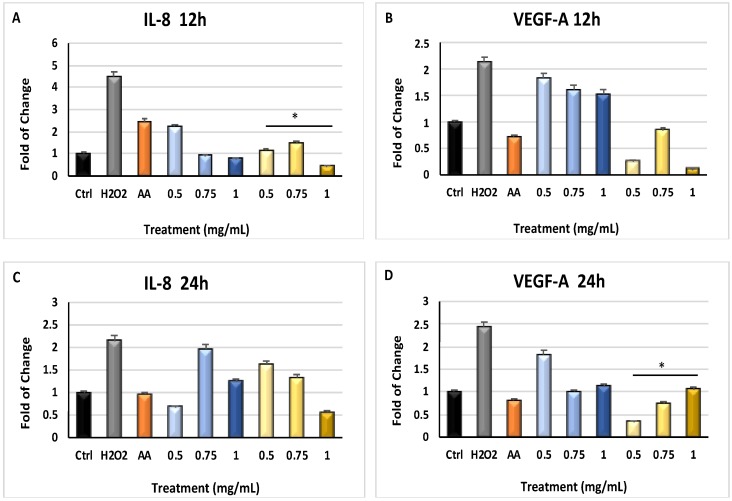
Gene expression of the proinflammatory cytokines Il-8 and VEGF-A. The expression of Interleukin 8 (IL-8) and Vascular endothelial growth factor A (VEGF-A) was evaluated at 12 h and 24 h (Panels (**A**) and (**B**), and panels (**C**) and (**D**), respectively). HFF1 were exposed to ascorbic acid (AA, orange bar), or to 0.5, 0.75 and 1 mg/ml seeds waste extracts (blue bars) or 0.5, 0.75 and 1 mg/mL pulp waste extracts (yellows bar). H_2_O_2_ (grey bar) represents HFF1 cells exposed to only H_2_O_2,_ without previous extracts treatment. The mRNA levels for each gene was expressed as fold of change (2^−∆∆Ct^) of mRNA levels observed in untreated HFF1 (CTRL, black bar) defined as 1 (mean ± SD; n = 6) and normalized to Glyceraldehyde-3-Phosphate-Dehidrogenase (GAPDH). Data are represented as mean ± SD referred to the control (* *p* ≤ 0.05, ** *p* ≤ 0.01 and *** *p* ≤ 0.001).

Figure 3 shows the effect of *Myrtus* extracts from industrial residual on VEGF-A and IL-8 gene expression. In particular, IL-8 mRNA levels were significantly downregulated (*p* ≤ 0.05) in cells pretreated with pulp or seeds extracts for 12 h (Panel A) and 24 h (Panel C), then exposed to H_2_O_2_, as compared to cells exposed to H_2_O_2_ without a pretreatment with the extracts. VEGF-A gene expression was lower in cells exposed to H_2_O_2_, after a culturing period of 12 h in the presence of the extracts, as compared to cells cultured without extracts (Panel B). This downregulation became significant when cells were pretreated with the extracts for 24 h (*p* ≤ 0.05) (Panel D).

### 2.5. Modulation of Cytochrome p450 CYP3A4 and CYP27B1 Gene Expression

**Figure 4:**
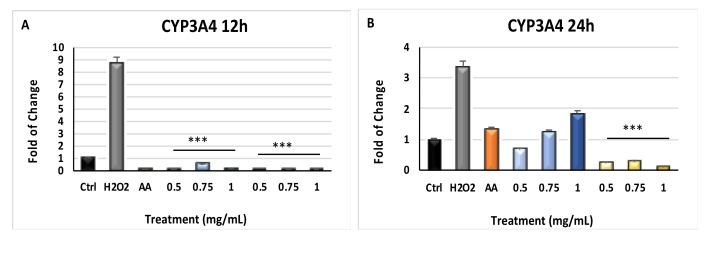
CYP3A4 gene expression. The expression of CYP3A4 was evaluated in cells cultured for 12 h and 24 h (Panels (**A**,**B**)) in the presence of different concentrations of pulp and seeds extracts, and then exposed to H_2_O_2_. HFF1 were exposed to ascorbic acid (AA, orange bar), or to 0.5, 0.75 and 1 mg/ml seeds waste extracts (blue bars) or 0.5, 0.75 and 1 mg/mL pulp waste extracts (yellows bar). H_2_O_2_ (grey bar) represents HFF1 cells exposed to only H_2_O_2,_ without previous extracts treatment. The mRNA levels for each gene was expressed as fold of change (2^−∆∆Ct^) of mRNA levels observed in untreated HFF1 (CTRL, black bar) defined as 1 (mean ± SD; n = 6) and normalized to Glyceraldehyde-3-Phosphate-Dehidrogenase (GAPDH). Data are represented as mean ± SD referred to the control (* *p* ≤ 0.05, ** *p* ≤ 0.01 and *** *p* ≤ 0.001).

**Figure 5:**
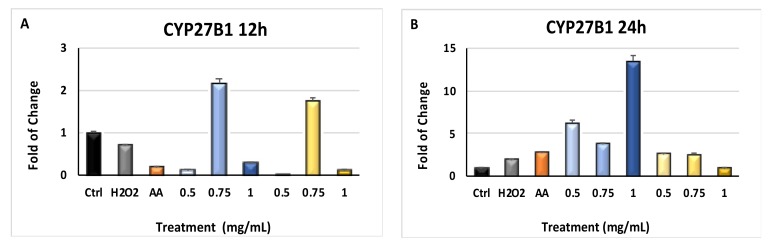
CYP27B1 gene expression. The expression of CYP27B1 was evaluated in cells cultured for 12 h and 24 h (Panels (**A**,**B**)) in the presence of different concentrations of pulp and seeds extracts, and then exposed to H_2_O_2_. HFF1 were exposed to ascorbic acid (AA, orange bar), or to 0.5, 0.75 and 1 mg/mL seeds waste extracts (blue bars) or 0.5, 0.75 and 1 mg/mL pulp waste extracts (yellows bar). H_2_O_2_ (grey bar) represents HFF1 cells exposed to only H_2_O_2,_ without previous extracts treatment. The mRNA levels for each gene was expressed as fold of change (2^yw^) of mRNA levels observed in untreated HFF1 (CTRL, black bar) defined as 1 (mean ± SD; n = 6) and normalized to Glyceraldehyde-3-Phosphate-Dehidrogenase (GAPDH). Data are represented as mean ± SD referred to the control (* *p* ≤ 0.05, ** *p* ≤ 0.01 and *** *p* ≤ 0.001).

Figure 4 shows the effects of *Myrtus* extracts on CYP3A4 and CYP27B1 gene expression. CYP3A4 (Figure 4, panels A and B) gene expression was significantly downregulated (*p* ≤ 0.001) in cells cultured in the presence of the extracts for 12 and 24 h, as compared to cells exposed to H_2_O_2_ without extracts pretreatment. CYP27B1 gene expression was also influenced by *Myrtus* exposure, showing an upregulation in cells exposed to H_2_O_2_ after 24 h of extracts-treatment, as compared to extracts-untreated cells (Figure 5, panel B).

## 3. Discussion

In general, skin and epithelial tissues represent the most important barrier to protect organisms from bacterial invasions and are also crucial in maintaining tissue homeostasis [43]. Chemical and physical stimuli, including UV radiations or oxidative stress, can influence skin structure and function, contributing to diseases such as photo-aging [44]. Within this context, ROS synthesis imbalance is involved in various skin disorders, including photosensitivity and other malignancies, starting from DNA damage events [45,46], which can be adjusted before the onset or during early phases by endogenous or external antioxidant supplements [47]. Several medicinal plants contain bioactive molecules, such as polyphenols and flavonoids, exhibiting pharmacological and medicinal properties against acute and chronic disorders through a protective action against free radical generation [48,49,50]. *Myrtus communis* L. is typical of Mediterranean area, has a high genetic diversity in population, and is known as ornamental plants for hydro-alcoholic infusions production and as medicament [51,52]. The medical potential of this herb is related to its antibacterial and analgesic properties, as well as anti-inflammatory and selective pro-apoptotic effects [53,54]. Here, we show that polyphenols found in *Myrtus* extracts were able to reduce the amount of ROS generated in our model (Figure 1), significantly inhibiting the expression of pr-inflammatory cytokines (Figure 2 and Figure 3).

Metabolism of P450 can lead to the formation of oxide radicals, being toxic and harmful for cells and tissues [19]. Polyphenols act inhibiting ROS production and CYPs activity, resulting in the prevention of ROS-induced DNA damages and suppression of cancer development, also improving the effect of oral drugs [25,48,55].

Other authors describe that extracts derived from different plants, exhibit an anticancer activity on different tumor cell lines, also modulating the expression and function of CYP3A4 [56,57,58]. Nevertheless, suppression of CYP3A4 levels in inflammatory diseases, can improve drug metabolism and value of therapeutic drugs for melanoma and psoriasis [59]. Our extracts inhibited CYP3A4 gene expression (Figure 4) and the related oxidative species production, considering that ROS represents one of the side effects of the CYP3A4 enzymatic mechanisms [60].

**Figure 6:**
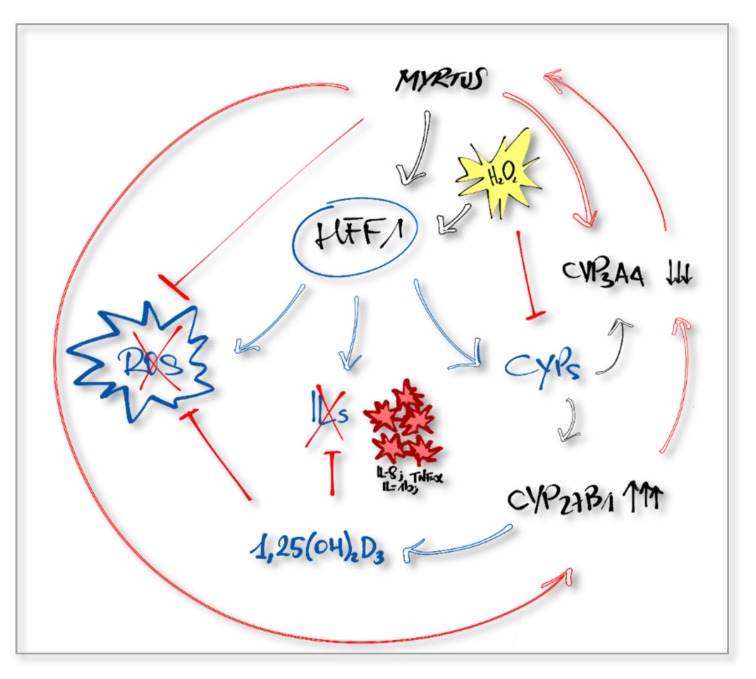
Regulatory activity of *Myrtus* products. *Myrtus* extracts exert antioxidant and anti-inflammatory activities against oxidative stress. They reinforce, acting in a synergic manner together with the physiological balancing systems and with other natural molecules, such as vitamin D.

CYP27B1 is another enzyme regulating metabolic reactions. It guides the conversion of cholecalciferol (vitamin D3) in its final active form, calcitriol (1,25(OH)_2_D_3_), through a hydroxylation reaction [24,61]. Activation and degradation of this molecule happens not only in the kidney, but also in other tissues, such as intestine, pancreas, immune cells, and skin [62]. Endogenous 1,25(OH)_2_D_3_ inhibits inflammatory pathways, modulating proinflammatory cytokines production by monocytes, macrophages, and fibroblasts, thus protecting tissues from hyperinflammatory response damages [18,63]. Normal healthy tissues show a high expression of CYP27B1, with stable levels of circulating active vitamin D [64]. Among the various functions, 1,25(OH)_2_D_3_ is required for epidermal differentiation, homeostasis, and maintenance of cutaneous permeability barrier [65]. Induction of oxidative stress in keratinocytes cultured in the presence of vitamin D in its active form promote the upregulation of CYP27B1 expression, leading to increased level of 1,25(OH)_2_D_3_ [66]. Here, we show that CYP27B1 gene expression is upregulated in fibroblasts treated with *Myrtus* extracts for 24 h, then exposed to H_2_O_2_ (Figure 5), as compared to cells not pretreated with extracts. The results could be related to a higher activation of 25(OH)_2_D_3_, most likely able to counteract the inflammatory response and oxidative stress induced by H_2_O_2_ treatment. Natural plant extracts display a large number of polyphenols and flavonoids, acting as antioxidant and anti-inflammatory molecules. These compounds can interact with drugs or natural molecules as vitamin D3, modifying their pharmacokinetic and regulating the expression of cytochromes P450 [67]. Therefore, natural extracts could exert a synergic antioxidant and anti-inflammatory action and potentiate the effects of other drugs, increasing their plasma concentration and improving their protective effect against oxidative stress induced damages [68]. Taken together, our results suggest that *Myrtus* could exert a synergic effect with endogenously synthesized 1,25(OH)_2_D_3_ in protecting cells from injuries related to impaired ROS-production (Figure 6). In conclusion, our findings disclose new strategies for the development of drugs for the prevention and treatment of inflammation caused by oxidative stress and chronic pathological inflammation states.

## 4. Materials and Methods

### 4.1. Reagents and Solvents

Standards of flavanol quercetin-3-O-galactoside, quercetin-3-O-rhamnoside, anthocyanins cyanidin-3-O-glucoside, petunidin-3-O-glucoside, peonidin-3-O-glucoside, malvidin-3-O-glucoside, and ellagic acid were purchased from Extrasynthese (Lyon, France). Gallic acid was purchased from Sigma (Milano, Italy). Ethanol absolute and acetonitrile were of analytical grade and were purchased from Sigma (Milano, Italy). Water was distilled and filtered through a Milli-Q apparatus (Millipore, Milan, Italy). Orthophosphoric acid were purchased from Carlo Erba (ACS ISO, for analysis, 85%).

#### 4.1.1. Preparation of the Biomass Extracts

The extracts used in this paper were obtained from the scraps of the myrtle liquor production. The myrtle marcs are the residual biomasses obtained from the infusion process of myrtle berries for the preparation of the liquor. At the end of the infusion process, the byproducts were separated from the hydro-alcoholic extract, pressed, and freeze-dried in order to easily separate pulp and seeds.

Samples were lyophilized and thinly pulverized in a home style coffee grinder, mixed thoroughly, and split up in three replicates. Of every powdered sample, 0.5 grams were added to 10 mL of a 70% (*v*/*v*) of ethanol aqueous solution in screw capped 15 mL Falcon tubes. The extraction was conducted in an ultrasonic bath for 60 min, under temperature control. The extract was centrifuged at 4000 rpm for 60 min at 20 °C and diluted with a 0.22 M aqueous solution of phosphoric acid, prior to its injection into the chromatographic system.

#### 4.1.2. Stock Standard Solutions of Polyphenols

A mix stock standard solution containing anthocyanins, flavanols, ellagic acid, and gallic acid was prepared in methanol at 1000 mg/L for all compounds. Intermediary standard solutions were prepared by diluting stock standard solution with the methanol and subsequently diluted with 0.22 M phosphoric acid to obtain mixed reference solutions in the range of 0.02–20 mg/L. All standard solutions were stored in the dark at −20 °C until usage.

#### 4.1.3. HPLC Analysis

Quali-quantitative analysis was carried out according to a methodology previously reported by Sarais et al. [69]. Briefly, a HPLC 1100 system (Agilent Technologies, Milan, Italy) equipped with a quaternary pump, a degasser, an auto-sampler, and a thermostat column compartment coupled with a DAD detector UV 6000 (Thermo Finnigan, Milan, Italy) was employed for separation and identification of main metabolites. The chromatographic separation was performed on a Kinetex column (5u, C18, 100 A, Phenomenex), operated with mobile phase A (acetonitrile) and B (H_2_O containing 0.22 M phosphoric acid), according to the following conditions: From 0 min (A:B 5:95, *v*/*v*) to 30 min (A:B 13:87, *v*/*v*), from 30 to 35min (A:B 15:85, *v*/*v*,) and finally from 35 to 70 min (A:B 30:70, *v*/*v*). A post-time of 15 min was used to allow the column to equilibrate before the next sample injections. The flow rate was 0.6 mL/min and the injection volume was 10 μL. The concentrations of the active ingredients were expressed as milligrams of active ingredient per 100 g of dry weight. All analyses were replicated three times. Data were expressed as the mean ± standard deviation (SD).

Quantification of the single polyphenols was performed using a five-point regression curve built with the available standards (see Supplementary Materials). Calibration was performed at the wavelength of the maximum UV–Vis absorbance (280 nm for gallic acid and derivatives, 360 nm for flavonols, and 520 nm for anthocyanins). Hydrolysable tannins amount was calculated using gallic acid as reference, moreover petunidin 3-glucoside was quantified using the calibration curve of malvidin 3-glucoside.

#### 4.1.4. Hydroxyl Radical Scavenging Activity

Electron Paramagnetic Resonance (EPR) spectroscopy, coupled with the spin trapping method, was used to assess the hydroxyl radical scavenging activity of the myrtle byproducts extracts. The hydroxyl radicals were generated in vitro by the Fenton reaction, in which a hydroxyl radical is generated through the oxidation of iron (II) to iron (III) by hydrogen peroxide [70]. The hydroxyl radical is trapped by the nitrone spin trap DMPO (5,5-Dimethyl-1-Pyrroline-N-Oxide) to form a DMPO-OH adduct that is detectable by EPR.

**Figure 7:**
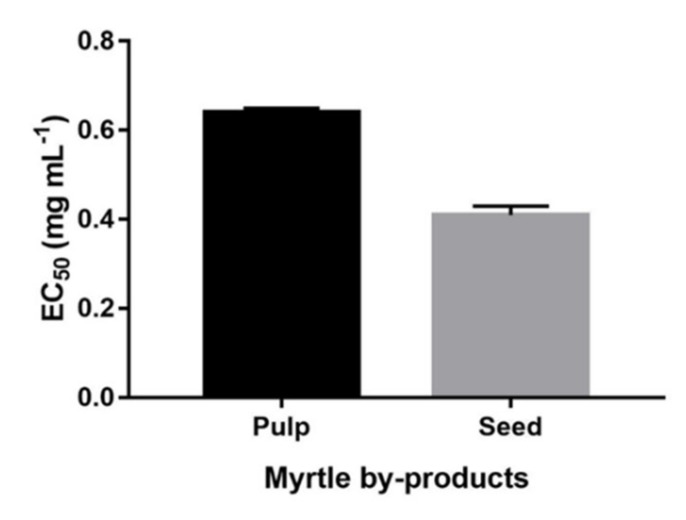
Hydroxyl radical scavenging activity of myrtle byproducts. Results are expressed as EC_50_ (n = 3).

The freeze-dried extracts were mixed with water in order to get the final concentration of 15 mg mL^−1^. Water extract with increasing concentrations was mixed with 0.1 mM iron (II) sulfate, DMPO 26 mM, and hydrogen peroxide 0.03% (*w*/*w*) to a final volume of 1 mL with water. The DMPO adduct was detected with a Bruker EMX spectrometer operating at the X-band (9.4 GHz) using a Bruker Aqua-X capillary cell. EPR instrument was set under the following conditions: modulation frequency, 100 kHz; modulation amplitude, 1 G; receiver gain, 1 × 105; microwave power, 20 mW. EPR spectra were recorded at room temperature immediately after the preparation of the reaction mixture. The concentration of the DMPO-OH adduct was estimated from the double integration of spectra. The percentage of inhibition was calculated against a blank with no extract applying the following formula:

100 × (A_0_ − A_s_)/A_0_ (1)

where A_0_ is the concentration of the spin adduct without extract and A_s_ is the concentration of the adduct after the reaction with the extract. Results were expressed as EC_50_ (Figure 7). Three replications were performed for each extract.

### 4.2. Cell Culturing Conditions

Human skin fibroblast 1 (HFF1) cells were purchased from (ATCC, Manassas, VA, USA) and cultured in a basic growing medium of DMEM low glucose (Life Technologies, Carlsbad, CA, USA), supplemented with 10% fetal bovine serum (FBS Life Technologies, Carlsbad, California, USA), 2 mM L-glutamine (Euroclone, Milano, Italy) and 1% of penicillin/streptomycin (Euroclone, Milano, Italy). Cells at passage 5 were then exposed to freeze-dried *Myrtus* extracts, previously suspended in cultured medium at three different concentrations of 0.5, 0.75 and 1 mg/mL, for 12 and 24 h. After treatment with the extracts, cells were incubated with 100 µM H_2_O_2_ in basic growing medium for 1 h, to induce oxidative stress. Other cells were cultured in growing medium without extracts, then exposed to H_2_O_2_ treatment (H_2_O_2_), representing our control of oxidative stress. HFF1 cultured in the presence of 100 µg/mL ascorbic acid (AA)(Sigma-Aldrich, Germany), then exposed to H_2_O_2,_ represent a positive control of antioxidant activity. Cells grown in basic medium alone, without any treatment, were considered the negative control (Ctrl).

### 4.3. MTT Viability Assay

Cytotoxicity of *Myrtus* extracts was evaluated by the MTT Thiazolyl Blue Tetrazolium Bromide assay (Sigma-Aldrich, Saint Louis, MO, USA). Cells were seeded at a concentration of 7000 cells/well in 96-well plates and cultured in the previous described conditions. After senescence induction with H_2_O_2_ treatment, the medium was removed, and cells were incubated for 2 h with 100 μl MTT at final concentration of 0.65 mg/mL. The formazan precipitated was then dissolved in DMSO and absorbance detected at 570 nm using Varian50 MPR, Microplate reader (Akribis Scientific, Common Farm, Frog Ln, Knutsford WA16 0JG, Great Britain). The results (data not shown) were compared to the untreated control group and viability of H_2_O_2_-senescent cells treated with extracts was calculated as:

% cell viability referred to untreated control cells = (OD_570_ treated cells) × 100 / (OD_570_ control). (2)

### 4.4. Measuring of ROS Production

To test the variation in ROS production by fibroblast after oxidative stress induction, carboxy-2′,7′-Dichlorofluorescin diacetate (H_2_DCFDA)(Sigma-Aldrich, Saint Louis, MO, USA) was used. After treatment with different *Myrtus* extracts, cells were incubated for 30 min with 1 μM carboxy-H_2_DCFDA in culture medium and then exposed to H_2_O_2_. ROS concentration was detected by spectrophotometric microplate reader using excitation and emission wavelength at 504 nm and 529 nm, respectively.

### 4.5. Gene Expression Analysis by Real Time-PCR

Total mRNA was isolated at time 0, 12, and 24 h from cells treated in previously described conditions using Trizol reagent (Life Technologies, Carlsbad, CA, USA) according to manufacture protocol. Approximately 1 µg of total RNA was reverse-transcribed and used for quantitative polymerase chain reaction according to the Power SYBR® Green RNA-to-CT™ 1-Step Kit protocol. Each sample was performed in triplicate under standard qRT-PCR conditions (48 °C for 30 min, 95 °C for 10 min, then cycled at 95 °C for 15 s and 60 °C for 1 min for 40 cycles) using a CFX Thermal Cycler (Bio-Rad, Hercules, CA, USA) (Applied Biosystems, Foster City, CA, USA). Target Ct values were normalized on hGAPDH, considered as a reference gene, while the mRNA levels of HFF1 treated in different conditions were expressed as fold of change (2^−∆∆Ct^) relative to the mRNA levels observed in HFF1 untreated cells. The qRT-PCR analysis was performed for the following genes: Interleukin 1 beta (IL-1β), Interleukin 8 (IL-8), tumor necrosis factor alpha (TNF-α), vascular endothelial growth factor A (VEGF-A), CYP3A4, and CYP27B1. All primers are described in Table 2.

### 4.6. Statistical Analysis

Statistical analysis was performed using Statistical Package for the Social Sciences version 13 Software (SPSS Inc., Chicago, IL, USA). Kruskal-Wallis rank sum was performed, assuming * *p* value ≤ 0.05, ** *p* ≤ 0.01 and ***p ≤ 0.001 as statistically significant. The experiments were conducted three times with three technical replicates for each treatment.

## 5. Conclusions

Extracts from *Myrtus* pulp and seeds, residues from industrial production, maintain their polyphenols content and their properties, exerting antioxidant and anti-inflammatory activities. Moreover, our extracts were also able to modulate CYP3A4 and CYP27B1 expression. Taken together, our results demonstrate, for the first time, that *Myrtus* polyphenols could interact with other molecules, enhancing the final therapeutic effects of drug treatments, by regulating normal metabolic reactions. This synergic effect could suggest a potential use of *Myrtus* extracts in controlling adverse effects of oxidative stress, thus preventing chronic inflammation, and define novel therapeutic strategies based on the implementation of currently used drug therapies by nutraceuticals.

## Figures and Tables

**Table 1:** Phenolic compounds quantified in myrtle liqueur byproducts. Average content (mg/kgDW) with their standard deviations, n = 3 replicates.

Analyte	Concentration mg/kg	
	Ethanol/Water	
	Pulp	Seeds
Gallic acid	52.2 ± 0.9	137.0 ± 6.8
Hydrolysable tannins ^a^	498.0 ± 20.5	11989.8 ± 205.2
Ellagic acid	350.5 ± 15.0	726.9 ± 28.3
*Flavonols*		
Quercetin-3-O-galactoside	191.0 ± 6.7	104.9 ± 9.3
Quercetin-3-O-rhamnosid	66.6 ± 3.0	62.0 ± 2.9
*Anthocyanins*		
Cyanidin-3-glucoside	1.8 ± 0.2	nd
Petunidin-3-glucoside ^b^	3.6 ± 0.3	nd
Peonidin-3-glucoside	13.5 ± 0.3	nd
Malvidin-3-glucoside	42.0 ± 2.4	nd

^a^ Expressed as gallic acid equivalent. ^b^ Expressed as malvidin-3-glucoside equivalent. Nd = no data

**Table 2:** Primers sequences.

Primers	Forward	Reverse
**hGAPDH**	GAGTCAACGGAATTTGGTCGT	GACAAGCTTCCCGTTCTCAG
**IL-1β**	GCTACGAATCTCCGACCACC	ATCGTGCACATAAGCCTCGT
**IL-8**	CTTCTCCACAACCCTCTG	GAACTGAGAGTGATTGAGAGT
**TNF-α**	CCTCAGACGCCACAT	GAGGGCTGATTAGAGAGA
**VEGF-A**	GCCAAGTGGTCCCAGGCTGC	TCGTCATTGCAGGCAGCCCCC
**CYP3A4**	TAGCCCAGCAAAGAGCAACA	CAAAAGGCCTCCGGTTTGTG
**CYP27B1**	CCTGAACCAGACCATGACCC	GAGCCTTTGCCATTCTTCGC

## Supplementary Materials

The following are available online, Figure S1: HPLC-DAD chromatogram of Myrtus by-products extracts, Table S1: Characteristics of phenolic compounds calibration curves.
