# Supplementary material for: Myrtus Polyphenols, from Antioxidants to Anti-Inflammatory Molecules: Exploring a Network Involving Cytochromes P450 and Vitamin D
Source: Molecules. 2019 Apr 17;24(8):1515. doi: 10.3390/molecules24081515 (PMC6515124; doi:10.3390/molecules24081515)
Supplement: Supplementary file 1 [file molecules-24-01515-s001.pdf]

Supplementary Materials

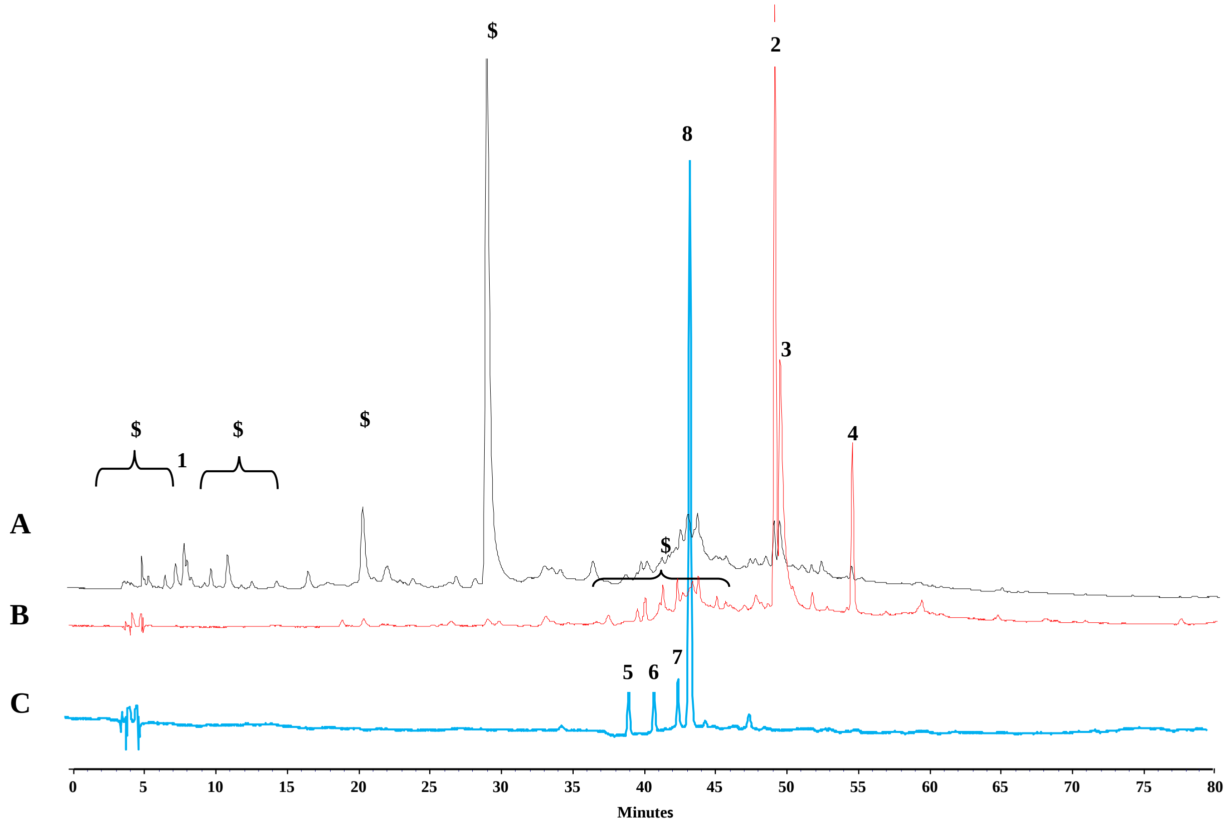

Figure S1. HPLC-DAD chromatogram at 280 nm (**A**), 360 nm (**B**) e 520 nm (**C**) of Myrtus by-products extracts, where (**1**) gallic acid, (**$**) hydrolizable tannins, (**2**) quercetin-3-O-galactoside, (**3**) ellagic acid, (**4**) quercetin-3-O-rhamnoside, (**5**) cyanidin 3-O-glucoside, (**6**) petunidin 3-O-glucoside, (**7**) peonidin 3-O-glucoside, (**8**) malvidin 3-O-glucoside.

Table 1. Characteristics of phenolic compounds calibration curves using the proposed method.

	Linearity range (mg/L)	Slope	Intercept	Correlation coefficient (r)
Gallic acid	0.02–20	476715	-15655	0.9994
Ellagic acid	0.05–10	199143	3023	0.9994
Quercetin-3-*O*-galactoside	0.05–10	289415	-10188	0.9996
Quercetin-3-O-rhamnoside	0.02–20	266692	6439.6	0.9991
Cyanidin-3-glucoside	0.02–20	586108	-29600	0.9990
Peonidin-3-glucoside	0.02–20	108539	-5491	0.9990
Malvidin-3-glucoside	0.02–20	431611	-18433	0.9992
